# Accuracy of pedicle screw insertion for unilateral open transforaminal lumbar interbody fusion: a side-by-side comparison of percutaneous and conventional open techniques in the same patients

**DOI:** 10.1186/s12891-020-3180-1

**Published:** 2020-03-14

**Authors:** Satoshi Sumiya, Fujiki Numano, Takahisa Ogawa, Toshitaka Yoshii, Atsushi Okawa, Hiromichi Komori

**Affiliations:** 1 Department of Orthopaedic and Spine Surgery, Yokohama-City Minato Red Cross Hospital, 3-12-1 Shinyamashita, Naka-ku, Yokohama City, Kanagawa 231-8682 Japan; 2 Department of Orthopaedic Surgery, Tokyo Medical and Dental University, Tokyo, Japan

**Keywords:** Unilateral open transforaminal lumbar interbody fusion, Percutaneous pedicle screw, Conventional open screw insertion

## Abstract

**Background:**

The aim of the study was to compare the accuracy of percutaneous pedicle screw (PPS) insertion (P-side) with that of conventional open screw insertion (O-side) during unilateral open transforaminal lumbar interbody fusion (TLIF) in the same patients. We also sought to determine the incidence of pedicle screw misplacement and to identify relevant risk factors.

**Methods:**

The study was a retrospective analysis of prospectively collected data for 766 pedicle screws placed in 181 consecutive patients who underwent a unilateral open-TLIF procedure in the lumbosacral spine. Our minimally invasive TLIF was performed by unilateral open freehand insertion of pedicle screws for decompression on one side and PPS on the opposite side. Using this approach, we were able to compare the accuracy of PPS insertion with that of conventional open screw insertion in the same patients. There were 383 PPSs and 383 screws inserted by the open method. The accuracy of screw placement was evaluated on reconstructed computed tomography images obtained postoperatively, and screw misplacement was classified. Potential risk factors for screw misplacement were investigated in three-level mixed-effects logistic regression analysis.

**Results:**

Thirty-four screws (8.9%) were misplaced on the P-side and 37 (9.5%) were misplaced on the O-side; the difference was not statistically significant (*P* = 0.803). Subclassification analysis revealed minor perforation of 28 screws (7.3%) on the P-side and 32 (8.4%) on the O-side, moderate perforation of 5 screws (1.3%) on the P-side and 4 (1.0%) on the O-side, and severe perforation of 1 screw (0.3%) on each side. Three-level mixed-effects logistic regression analysis identified body mass index as a significant risk factor for screw misplacement on the P-side (odds ratio 1.194, 95% confidence interval 1.066–1.338).

**Conclusions:**

Accuracy of pedicle screw insertion was not significantly different between PPS insertion and conventional open screw insertion in the same patients. Body mass index had a significant influence on the risk of screw misplacement in PPS insertion.

## Background

Minimally invasive transforaminal lumbar interbody fusion (MIS-TLIF) has been well described in the literature [1–4] and is widely performed in patients with lumbar spinal disease. Pedicle screws are used for MIS-TLIF but must be inserted correctly because misplacement can lead to devastating complications. Some previous studies have investigated the accuracy of pedicle screw insertion [5, 6] and compared percutaneous pedicle screw (PPS) insertion with insertion using the conventional open technique [7, 8]. These reports indicate wide variation in the accuracy of pedicle screw insertion. Some studies have found that the accuracy of PPS insertion is better than that of conventional open insertion [9–12], but other studies have found no significant difference [7, 8]. However, these studies did not examine the techniques through side-by-side comparison in the same patients. Furthermore, several causes of pedicle screw misplacement have been identified [5, 6].

At our institution, we perform unilateral open TLIF using unilateral open freehand insertion of pedicle screws for decompression on one side and PPS insertion on the contralateral side. This aim of this study was to compare the accuracy of PPS insertion with that of conventional open insertion in the same patients. We also sought to determine the incidence of pedicle screw misplacement and to identify relevant risk factors.

## Methods

### Patients

This retrospective study involved 181 consecutive patients (91 men, 90 women; mean age 69.0 ± 10.9 years at the time of surgery) who underwent single-level (*n* = 160) or double-level (*n* = 21) unilateral open-TLIF for lumbar spinal canal stenosis with degenerative spondylolisthesis and degenerative instability between April 2011 and March 2016 at Yokohama City Minato Red Cross Hospital. Mean body mass index was 24.1 ± 3.7. The patients were evaluated preoperatively by magnetic resonance imaging (MRI) and computed tomography (CT). No patient was excluded. A total of 766 pedicle screws placed in the lumbosacral spine, of which 383 were PPS (P-side) and 383 were pedicle screws inserted by the conventional open method (O-side). Two screws were placed at L2, 47 at L3, 141 at L4, 145 at L5, and 48 at S1 (Table 1).

**Table 1:** Characteristics of patients

Variables		P-side		O-side
Number of patients			181	
Age (year ± SD)			69.0 ± 10.9	
Gender	male		91	
	female		90	
BMI (m/kg^2^ ± SD)			24.1 ± 3.7	
Fusion level	1-level		160	
	2-level		21	
Pedicle screw level (No. of screw)				
L2		2		2
L3		47		47
L4		141		141
L5		145		145
S1		48		48
Total		383		383
Screw total			766	

*PPS* Percutaneous Pedicle screw, *SD* Standard deviation, *BMI* Body Mass Index, *No* Number, *P-side* PPS side, *O-side* Open side

The study was approved by the Yokohama City Minato Red Cross Hospital Research Ethics Committee (approval number 2018–77).

### Surgical procedure

**Fig. 1:**
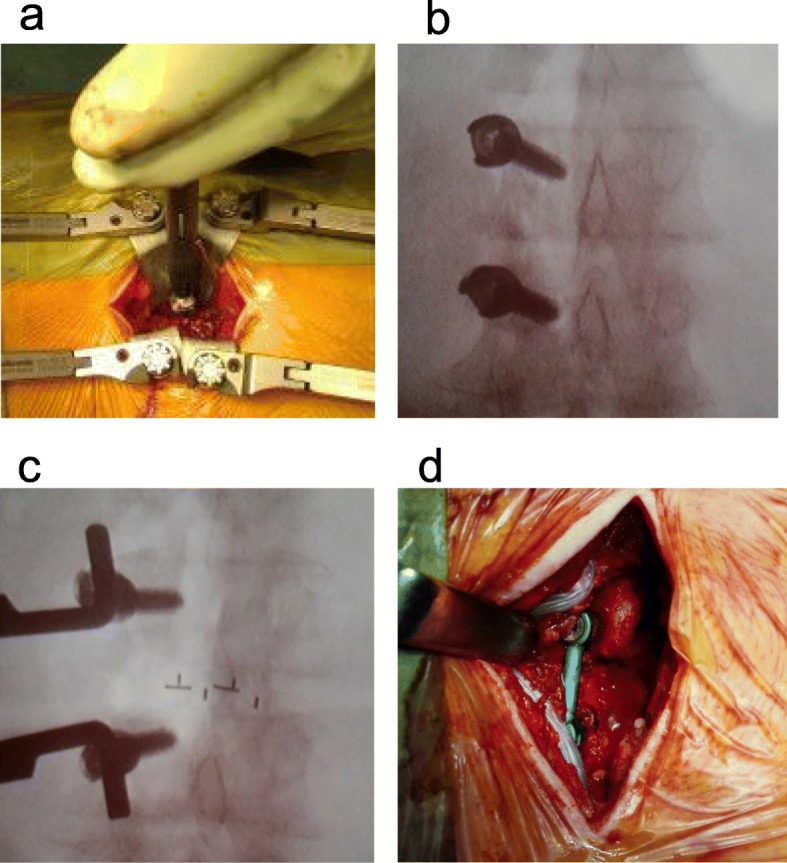
Pedicle screws were inserted using the conventional open technique. **a** Pedicle screws were inserted into the pedicles on the open side. **b** Pedicle screws were inserted under a fluoroscopic axial view. **c** Bone grafts and two cages were packed into the disc space on a fluoroscopic axial view. **d** Connection of the rod

The patient was placed in the prone position with the trunk on a Relton-Hall frame. Following induction of anesthesia, standard surgical exposure was performed, with a 5-cm midline skin incision and unilateral exposure of the transverse processes. The open side was decided based on the patient’s symptoms. The base of the transverse process was perforated using an air drill, and marking wires (diameter, 2.0 mm) were inserted into the pedicle. After the positions of the marking wires were confirmed using C-arm fluoroscopy, the wires were removed and the pedicles were tapped using the freehand technique. Pedicle screws were then inserted into the pedicles using the freehand method. We performed unilateral laminectomy, bilateral decompression for central stenosis, and foraminal decompression for foraminal stenosis using microscopy. After decompression, bone grafts and 2 cages were packed into the disc space. The wound was closed in layers (Fig. 1).

**Fig. 2:**
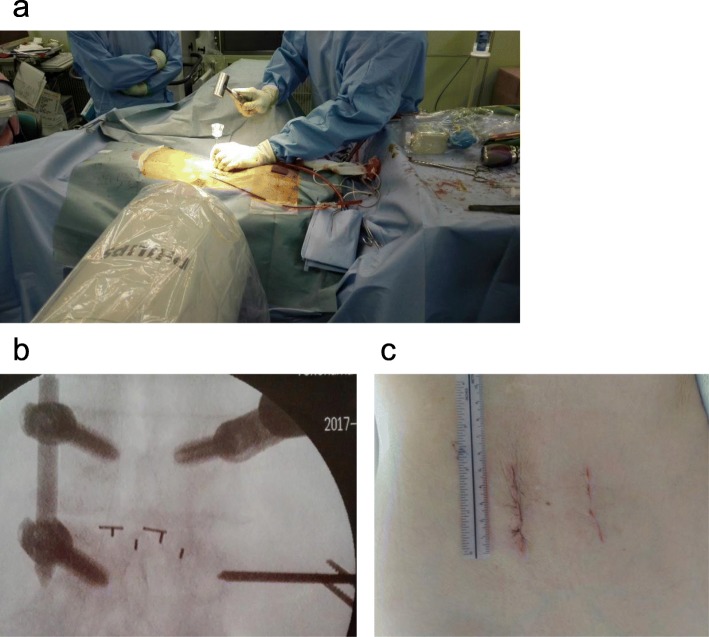
Percutaneous pedicle screw insertion. **a** The targeting needle is inserted into the pedicle under C-arm fluoroscopic guidance. **b** The targeting needle is inserted on a fluoroscopic axial view. **c** Surgical trace

Next, a 1.5-cm stab incision was made laterally on the side opposite to the open side. The targeting needle was then inserted into the pedicle at the superolateral border based on the anterior-posterior view under C-arm fluoroscopic guidance. The guidewire was inserted through the targeting device and into the pedicle. The pedicle was tapped using the guidewire. Finally, the pedicle screw was inserted over the guidewire. Other PPSs were inserted using the same method (Fig. 2). The size of the screws was the same on both sides in all patients. Some screw insertions were performed by spine surgeons and others by residents.

### Evaluation of screw position

**Fig. 3:**
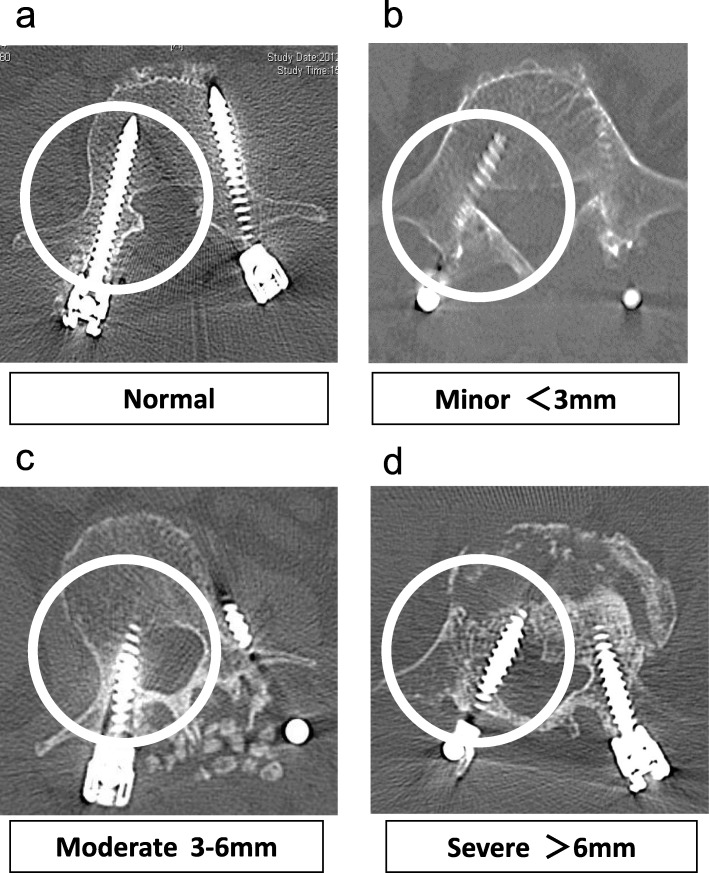
Evaluation of pedicle screw misplacement. **a** Normal. **b** Minor perforation (< 3 mm). **c** Moderate perforation (3–6 mm). **d** Severe perforation (> 6 mm)

Postoperative CT scans were obtained using a 16-row multidetector CT system (Canon Medical Systems, Tokyo, Japan) to assess the implant position in all patients. The series consisted of 3.0-mm CT sections that were reconstructed at 2.0-mm intervals. A spine surgeon not otherwise involved in the study evaluated the position of the screw in the pedicle wall in the axial, sagittal, and coronal planes. Screw misplacement was defined using the system proposed by Schizas et al. [13] as minor (< 3 mm), moderate (3–6 mm), or severe (> 6 mm). The direction of the perforation was defined as medial, lateral, inferior, or superior (Fig. 3).

### Statistical analysis

Statistical analysis was performed using Fisher’s exact test and the Mann-Whitney *U* test. To identify risk factors for misplacement, three-level mixed-effects logistic regression analysis was performed that accounted for possible clustering of screws (level 1) nested within the vertebral level (level 2), and the vertebral nested within the individual (level-3). We adjusted for age, sex, and BMI and the operated side and level. Statistical analysis were performed using the STATA version 16.0 (Stata Corp, College Station, TX, USA) and the commercial package JMP Version 13.1.0 software (SAS Institute Inc., Cary, NC, USA). A *P*-value < 0.05 was considered statistically significant.

## Results

A total of 766 pedicle screws (383 on the P-side, 383 on the O-side) were inserted in 181 patients. The number of successfully placed pedicle screws was 349 (91.1%) on the P-side and 346 (90.5%) on the O-side. Screw misplacement was observed for 34 screws (8.9%) on the P-side and 37 (9.5%) on the O-side; the difference was not statistically significant (*P* = 0.803). Subclassification analysis revealed minor perforation of 28 screws (7.3%) on the P-side and 32 (8.4%) on the O-side; moderate perforation of 5 screws (1.3%) on the P-side and 4 (1.0%) on the O-side, and severe perforation of 1 screw (0.3%) on each side. The direction of misplacement was inferior for 2 screws on the P-side and 1 screw on the O-side, superior for 2 screws on the P-side and 1 screw on the O-side, medial for 22 screws on the P-side and 23 screws on the O-side, and lateral for 8 screws on the P-side and 12 screws on the O-side. Screw misplacement occurred at the following vertebral levels: L2 for 1 screw on the P-side and 1 screw on the O-side, L3 for 3 screws on the P-side and 5 screws on the O-side, L4 for 18 screws on the P-side and 15 screws on the O-side, L5 for 8 screws on the P-side and 15 screws on the O-side, and S1 for 4 screws on the P-side and 1 screw on the O-side. Four screws on the P-side and 2 screws on the O-side showed moderate or severe penetration medially. One screw on the P-side and 1 screw on the O-side were associated with neurological symptoms and therefore were replaced in both cases (Table 2).

**Table 2:** Comparison of distribution for pedicle screw misplacement in the PPS side and the open side

Categorical variable		P-side	O-side	*P*-value
Adequate insertion (%)		349 (91.1)	346 (90.5)	
Misplacement (%)		34 (8.9)	37 (9.5)	NS
Penetration (%)	Minor	28 (7.3)	32 (8.4)	NS
	Moderate	5 (1.3)	4 (1.0)	NS
	Severe	1 (0.3)	1 (0.3)	NS
Direction	Inferior	2	1	NS
	Superior	2	1	NS
	Medial	22	23	NS
	Lateral	8	12	NS
Level	L2	1	1	NS
	L3	3	5	NS
	L4	18	15	NS
	L5	8	15	NS
	S1	4	1	NS

*NS* nonsignificant, *P-side* PPS side, *O-side* Open side

There was no statistically significant difference in the frequency of screw misplacement between the P-side and O-side according to age or sex, operated side, or fixed level. BMI was significantly higher in the group with screw misplacement on the P-side (Table 3). On the O-side, there was no significant difference in age or sex, operated side, fixed level, or BMI between the group with accurately placed screws and the group with misplaced screws. Furthermore, there was no significant difference in the accuracy of screw placement on the P-side according to age or sex, operated side, or fixed level. However, BMI was significantly higher in the group with screw misplacement on the P-side (Table 4). BMI was the only significant risk factor for screw misplacement identified as significant in the three-level mixed effect logistic regression analysis. The odds ratio of screw misplacement for obesity was 1.194 (95% confidence interval, 1.066–1.338) on the P-side (Table 5). However, BMI was not found to be a risk factor on the O-side.

**Table 3:** Comparison of variables for pedicle screw misplacement in the PPS side and the open side

Categorical variable		P-side	O-side	*P*-value
Age (year ± SD)		69.2 ± 10.8	72.1 ± 8.9	NS
Gender	male	13	17	NS
	female	19	16	NS
Side	Right	14	16	NS
	Left	19	21	NS
Fixed level	single	27	30	NS
	double	5	3	NS
BMI* (m/kg^2^ ± SD)		26.3 ± 4.0	24.1 ± 3.6	*P* = 0.027

*SD* Standard deviation, *BMI* Body Mass Index, *NS* nonsignificant, *P-side* PPS side, *O-side* Open side

*P < 0.05

**Table 4:** Comparison of the normal group and the screw misplacement group in each side

Side	Categorical variable		Normal group	Misplacement group	*P*-value
P-side	age		69.1	69.1	NS
	BMI*		23.7	26.3	*P* = 0.001
	Gender	male	78	13	
		female	71	19	NS
	Inserted side	Right	66	14	
		Left	83	18	NS
	Fixed level	1	133	27	
		2	16	5	NS
O-side	age		68.5	72.2	NS
	BMI		24.1	24.1	NS
	Gender	male	74	17	
		female	74	16	NS
	Inserted side	Right	87	14	
		Left	61	19	NS
	Fixed level	1	130	30	
		2	18	3	NS

*BMI* Body Mass Index, *NS* nonsignificant, *P-side* PPS side, *O-side* Open side

*P < 0.05

**Table 5:** Fixed effects of risk factors for screw misplacement from the three-level mixed effect logistic regression

side	Risk factor	Odds ratio	95% CI	*P*-value
P-side	BMI	1.194	(1.066–1.338)	*P* = 0.002
O-side	–	–	–	–

Stratified by PPS or Open surgery

Model Adjusted for age, sex, operation side, BMI, and operation levels

## Discussion

Many authors have reported that MIS-TLIF can reduce tissue damage, blood loss, and postoperative pain compared with conventional TLIF [1–4]. In particular, PPS insertion can decrease damage not only to soft tissue but also to the superior facet joint because it is easier for surgeons to insert pedicle screws from the outside of the facet joint [14]. However, PPS insertion requires fluoroscopy or a navigation system.

Screw misplacement rates in the range of 8.8–23% have been reported for PPS insertion with C-arm fluoroscopy [5, 15, 16] while rates of 8.9–31% have been found for conventional open insertion [11, 17, 18]. Furthermore, the screw misplacement rate when using a navigation system has been reported to be 8–19% with fluoroscopy and 0–11% with CT [11].

In our study, the perforation rate was 8.9% for PPS insertion and 9.5% for conventional open insertion. Many studies have reported high accuracy of pedicle screw insertion. Similarly, the accuracy of PPS insertion was as high as that of conventional open insertion in our study.

In addition, some authors have reported that placement accuracy is not significantly different between PPS insertion and conventional open insertion [7, 8]. Similarly, we found no statistically significant difference in accuracy between PPS insertion and conventional open insertion.

Pedicle screw misplacement may lead to serious complications, such as neurovascular injury [19–22]. Therefore, an advanced technique for pedicle screw insertion is needed. As described above, navigation-assisted pedicle screw insertion is the most accurate. However, a navigation system is expensive and requires time for registration of anatomic landmarks. Therefore, there is a need to improve the precision of pedicle screw insertion techniques without use of a navigation system and to investigate the causes of pedicle screw misplacement.

Previous studies have identified intraoperative and preoperative factors that increase the likelihood of pedicle screw deviation, such as a thoracic level, deformity, obesity, and older age [5, 6, 23]. Our study involved only patients with lumbar spine pathology, and the success of single- or double-level fusion is not affected by deformity.

Several authors have reported that obesity is a significant risk factor for pedicle screw misplacement [5, 6, 24]. Consistent with these previous reports, we found that the likelihood of pedicle screw deviation was affected by BMI. However, in our study, BMI was only a significant risk factor for misplacement in the PPS group. A previous study found BMI to be a significant risk factor for screw misplacement with conventional open insertion [6] and other reported that BMI was a significant risk factor in PPS insertion [5]. Therefore, although these are isolated reports, both identified BMI as a risk factor. In our study, in which both insertion techniques were performed in the same patients, BMI was again a significant risk factor but only for PPS.

**Fig. 4:**
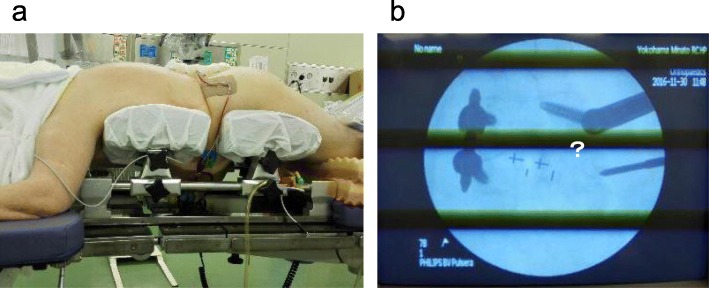
A representative clinical case of pedicle screw insertion in an obese patient. **a** A 71-year-old woman with a body mass index of 32 placed in the prone position. **b** Visualization of the L5 pedicle on the fluoroscopic axial view is blurred on the right side

Our method of PPS insertion requires C-arm fluoroscopy. In obese patients, the pedicle is shielded from some irradiation in C-arm fluoroscopy. As a result of the smaller radiation dose, visualization of the pedicle is difficult and intraoperative C-arm fluoroscopic and radiographic discrimination of anatomical characteristics is hindered by blurred visibility. A representative clinical case of obesity is shown in (Fig. 4a). The patient was a 71-year-old woman with a BMI of 32 who underwent spinal fusion for lumbar spinal canal stenosis. Unilateral open-TLIF was performed at L4/5, and the intraoperative C-arm fluoroscopic view of the L5 pedicle was blurred on the right side (Fig. 4b). Furthermore, the surgical procedure was performed at a deeper point with limited visualization. Kim et al. reported that the volume of the multifidus muscle was also a significant risk factor for pedicle screw misplacement and inferred that fluoroscopic images are often blurred in areas where the tissue is bulky [5].

In our study, older age tended to be a risk factor when using conventional open insertion (*P* = 0.086), likely because bone (especially cortical bone) becomes more fragile with aging and is more easily perforated. Furthermore, Oh et al. reported that bone density was an important factor in pedicle wall penetration. The penetration rate was higher in patients with stronger bones in whom the direction of screw insertion was difficult to modify. However, the trajectory can easily be changed during the procedure in weak bone [7]. It has also been observed that there is a learning curve for the accuracy of pedicle screw placement [25]. We believe that the accuracy of pedicle screw insertion depends on the surgeon’s technique when using conventional open insertion.

This study has several limitations. First, some of the insertion procedures were performed by spine surgeons and others by residents. Second, the study had a retrospective single-center design. However, the position of the screw was assessed by a spine surgeon who was not otherwise involved in the study and a 16-row multidetector CT system was used to confirm the implant position in all patients.

## Conclusions

This is the first study to compare the accuracy of pedicle screw insertion between PPS insertion and conventional open insertion in the same patients. There was no significant difference in accuracy between the two techniques. BMI was found to be a significant risk factor for screw deviation with PPS insertion but not with conventional open insertion. These findings suggest that the accuracy of PPS insertion is affected by BMI because this method relies heavily on radiologic imaging and that PPS insertion is thus not optimal in obese patients.

## Data Availability

The data and materials may be made available upon request by sending an e-mail to the first author.

## Abbreviations

CT: Computed tomography
MIS: Minimally invasive surgery
MRI: Magnetic resonance imaging
PPS: Percutaneous pedicle screw
TLIF: Transforaminal lumbar interbody fusion
